# Plasma metagenomic next-generation sequencing of microbial cell-free DNA detects pathogens in patients with suspected infected pancreatic necrosis

**DOI:** 10.1186/s12879-022-07662-2

**Published:** 2022-08-05

**Authors:** Donghuang Hong, Peng Wang, Jingzhu Zhang, Kaiwei Li, Bo Ye, Gang Li, Jing Zhou, Zhihui Tong, Lu Ke, Songjing Shi, Weiqin Li

**Affiliations:** 1 The First School of Clinical Medicine, Southern Medical University, Guangzhou, China; 2 Department of Critical Care Medicine, Fujian Provincial Hospital, No.134 East Street, Fuzhou, 350001 Fujian China; 3 Center of Severe Acute Pancreatitis (CSAP), Department of Critical Care Medicine, Jinling Hospital, Medical School of Nanjing University, No. 305 Zhongshan East Road, Nanjing, 210002 Jiangsu China; 4 National Institute of Healthcare Data Science, Nanjing University, Nanjing, China

**Keywords:** Infected pancreatitis necrosis, Acute pancreatitis, Metagenomic next-generation sequencing (mNGS), Microbial cell-free DNA, Pathogen detection

## Abstract

**Background:**

Infected pancreatic necrosis (IPN) is a life-threatening complication of acute pancreatitis (AP). Timely diagnosis of IPN could facilitate appropriate treatment, but there is a lack of reliable non-invasive screening tests. In this study, we aimed to evaluate the diagnostic value of plasma metagenomic next-generation sequencing (mNGS) based on circulating microbial cell-free DNA in patients with suspected IPN.

**Methods:**

From October 2020 to October 2021, 44 suspected IPN patients who underwent plasma mNGS were reviewed. Confirmatory diagnosis of IPN within two weeks after the index blood sampling was considered the reference standard. The confirmation of IPN relied on the microbiological results of drains obtained from the necrotic collections. The distribution of the pathogens identified by plasma mNGS was analyzed. Positive percent agreement (PPA) and negative percent agreement (NPA) were evaluated based on the conformity between the overall mNGS results and culture results of IPN drains. In addition, the clinical outcomes were compared between mNGS positive and negative patients.

**Results:**

Across all the study samples, thirteen species of bacteria and five species of fungi were detected by mNGS. The positivity rate of plasma mNGS was 54.55% (24/44). Of the 24 mNGS positive cases, twenty (83.33%, 95% CI, 68.42–98.24%) were consistent with the culture results of IPN drains. The PPA and NPA of plasma mNGS for IPN were 80.0% (20/25; 95% CI, 64.32–95.68%) and 89.47% (17/19; 95% CI, 75.67–100%), respectively. Compared with the mNGS negative group, patients in the positive group had more new-onset septic shock [12 (50.0%) vs. 4 (20.0%), p = 0.039].

**Conclusion:**

IPN relevant pathogens can be identified by plasma mNGS, potentially facilitating appropriate treatment. The clinical application of mNGS in this cohort appears feasible.

**Supplementary Information:**

The online version contains supplementary material available at 10.1186/s12879-022-07662-2.

## Background

Acute pancreatitis (AP) is a common gastrointestinal disease requiring hospitalization worldwide [1]. Infected pancreatic necrosis (IPN) and its related sepsis contribute substantially to morbidity and mortality in AP patients [2–4]. Microbiological evidence from (peri)pancreatic drains is the gold standard for IPN diagnosis, but invasive procedures are required, and the sensitivity is not satisfactory [5]. Unique radiological findings are an alternative to confirm IPN, but it also has low sensitivity without information regarding specific pathogens [6]. Due to the limitation of the current diagnostic approaches, the diagnosis of IPN largely rests on clinical symptoms and signs lacking specificity [7, 8]. Therefore, a reliable and non-invasive diagnostic approach is of great clinical value in AP patients with infection-like symptoms.

The diagnostic difficulty would potentially lead to irrational or prolonged use of antibiotics and unnecessary invasive procedures. Studies have shown that bacteremia was an independent risk factor for IPN [9, 10]. However, blood culture has low sensitivity and is time-consuming. Alternatively, metagenomic next-generation sequencing (mNGS) is a high-throughput sequencing method that can directly detect the nucleic acids of pathogens in clinical specimens, which is known for its short detection cycle and high sensitivity [11, 12]. Compared with culture, mNGS can improve the sensitivity and specificity in the diagnosis of bloodstream infection [13, 14]. The mNGS technology based on circulating microbial cell-free DNA (mcfDNA) can comprehensively identify pathogens causing infection anywhere in the body [15]. Plasma mNGS has also been applied to diagnose multiple infectious diseases, including invasive fungal infections, tuberculosis, and endocarditis [16–18].

The role of circulating mcfDNA in IPN patients is rarely explored. This study aimed to evaluate the diagnostic value of plasma mNGS tests in patients with suspected IPN.

## Methods

### Study design and ethics

This is a retrospective database-based cohort study conducted in Nanjing Jinling Hospital. The establishment of the database was approved by the institutional ethics committee of Nanjing Jinling Hospital (2019NZKY009-01). Broad informed consent was obtained from each participant on using the clinical and laboratory data for academic research. The clinical and laboratory data were collected and stored in a web-based electronic database (Acute Pancreatitis Database).

### Patient selection

Adult subjects diagnosed with AP admitted to the center of severe acute pancreatitis (CSAP), Jinling Hospital (Nanjing, China) from October 2020 to October 2021 were screened. The diagnosis and severity of AP were defined according to the revised Atlanta classification 2012 [19].

The inclusion criteria were as follows: (1) Plasma mNGS was performed when IPN was suspected but not yet confirmed. Suspected IPN was based on clinical manifestations like fever with elevated inflammatory markers. The decision for an mNGS test is made by the treating physician. (2) Sampling from AP onset < 35 days. (3) Survived more than 14 days after sampling. The exclusion criteria were pregnancy and confirmed extra-pancreatic infectious complications at screening.

### Metagenomic next-generation sequencing and analysis

Whole blood samples (3–5 ml) were sent for PACEseq mNGS analysis (Hugobiotech, Beijing, China). The human cells of each sample were removed by centrifugation. The supernatant was collected for DNA extraction using TIANGEN DP316 kit (TIANGEN, Beijing, China) based on its manual. NEBNext Ultra II DNA library Prep Kit (NEB, Ipswich, UK) was then used to construct the DNA libraries according to the manufacturer’s instructions. The quality of all libraries was measured by Qubit (Thermo Fisher Scientific, MA, USA) and Agilent 2100 Bioanalyzer (Agilent Technologies, Palo Alto, USA). The qualified libraries were finally sequenced on a Nextseq 550 platform (Illumina, San Diego, USA). Positive and negative controls were set for each batch during the experiments. The raw data were analyzed on PACEseq (Hugobiotech, Beijing). Adapters, as well as low quality, low-complexity, and short reads (less than 35 bp) were removed from the raw data. SNAP and Burrow-Wheeler alignment was then applied to exclude human sequences by mapping the reads to the human reference genome (hg38). The screened sequences were finally mapped to the microorganisms (bacteria, viruses, fungi, protozoa, and other multicellular eukaryotic pathogens) genome data registered with the NCBI Refseq database (https://ftp.ncbi.nlm.nih.gov/genomes/refseq/). All parameters of the detected pathogenic microorganisms were calculated, including the sequence reads, relative abundance, genome coverage, and depth. The reads number and reads per million (RPM) of each detected pathogen were calculated. For detected microbes, including bacteria (*Mycobacteria* excluded), and fungi (*Cryptococcus* excluded), a positive mNGS result was given when its coverage ranked top10 of the same kind of microbes and was absent in the negative control (“no template” control, NTC) or when its ratio of RPM between sample and NTC (RPM_sample_/RPM_NTC_) > 10 if RPM_NTC_ ≠ 0. For *M. tuberculosis*, and *Cryptococcus*, a positive mNGS result was considered when at least 1 unique read was mapped to species level and absent in NTC or RPM_sample_/RPM_NTC_ > 5 when RPM_NTC_ ≠ 0 [20]. Since the virus sequence was not verified by polymerase chain reaction (PCR), the cases in which only the virus sequence was detected were defined as mNGS negative.

### Microbial culture

Blood samples of all patients were sent for microbial culture at the same time as the mNGS test was performed. The blood samples were analyzed using the blood culture (BC) instrument BD BACTECTMFX40 (Becton Dickinson) according to the manufacturer’s manual. Positive blood culture samples were speciated using the Vitek MS system (BioMerieux, version 1.7, France).

Pathogens are classified according to Gram-negative bacteria, Gram-positive bacteria, and fungus. Polymicrobial infection was defined as more than one pathogen detected in a sample.

### Diagnosis of confirmatory IPN

In this study, confirmatory diagnosis of IPN within two weeks after sampling was considered the reference standard. IPN was confirmed when a positive microbial culture was obtained from (peri)pancreatic drains through percutaneous fine-needle aspiration or during drainage procedures and/or operative necrosectomy. Otherwise, sterile pancreatic necrosis (SPN) would be defined. The decision of invasive intervention is decided by the treating physician.

### Clinical outcome and definitions

Clinical outcome measures include in-hospital mortality, length of hospital stay (LOS), requirement of ICU admission, new-onset organ failure based on the modified Marshall’s score [19], new-onset sepsis, and septic shock defined according to the SEPSIS 3.0 definitions [21], management-related measures, and gastrointestinal fistula or abdominal bleeding requiring invasive intervention [22]. ‘New-onset’ in this study was defined as events that occurred after sampling and were not present 24 h before sampling.

### Data extraction

Data were extracted using a data extraction form developed in advance. Data concerning demographic and baseline clinical characteristics, including age, gender, etiologies, laboratory biochemistry, and clinical scores like Acute Physiology and Chronic Health Evaluation II (APACHE II) score, Computed Tomography (CT) severity index, and sequential organ failure assessment (SOFA) score at screening were extracted from the database. For identification of the pathogens, mNGS results were collected based on the standard reports. Before analysis, cross-checking was done on the data by the principal investigators to ensure the quality of the data.

### Statistical analysis

The overall results of plasma mNGS tests will be adjudicated as true positive (TP), false positive (FP), true negative (TN), or false negative (FN), as reported in previous studies [23, 24]. The overall result was considered as true positive if the plasma mNGS detected at least one IPN-relevant organism, while it was considered as false positive if the plasma mNGS detected pathogens that were not in accordance with the IPN diagnoses. We used the abovementioned reference standard to estimate positive percent agreement (PPA) and negative percent agreement (NPA). Results were reported as percentages with 95% CI [25].

Continuous variables were reported as the median with interquartile range (25%, 75%). Categorical variables were expressed in frequencies and percentages. Mann–Whitney U tests were used to compare the differences between the groups for continuous variables. Fisher’s exact tests were used for comparing categorical variables. SPSS 26 and Graphed Prism7 software have been applied for data analysis. All tests were two-tailed, and p-values of less than 0.05 were considered statistically significant.

## Result

### Baseline characteristics

**Fig. 1:**
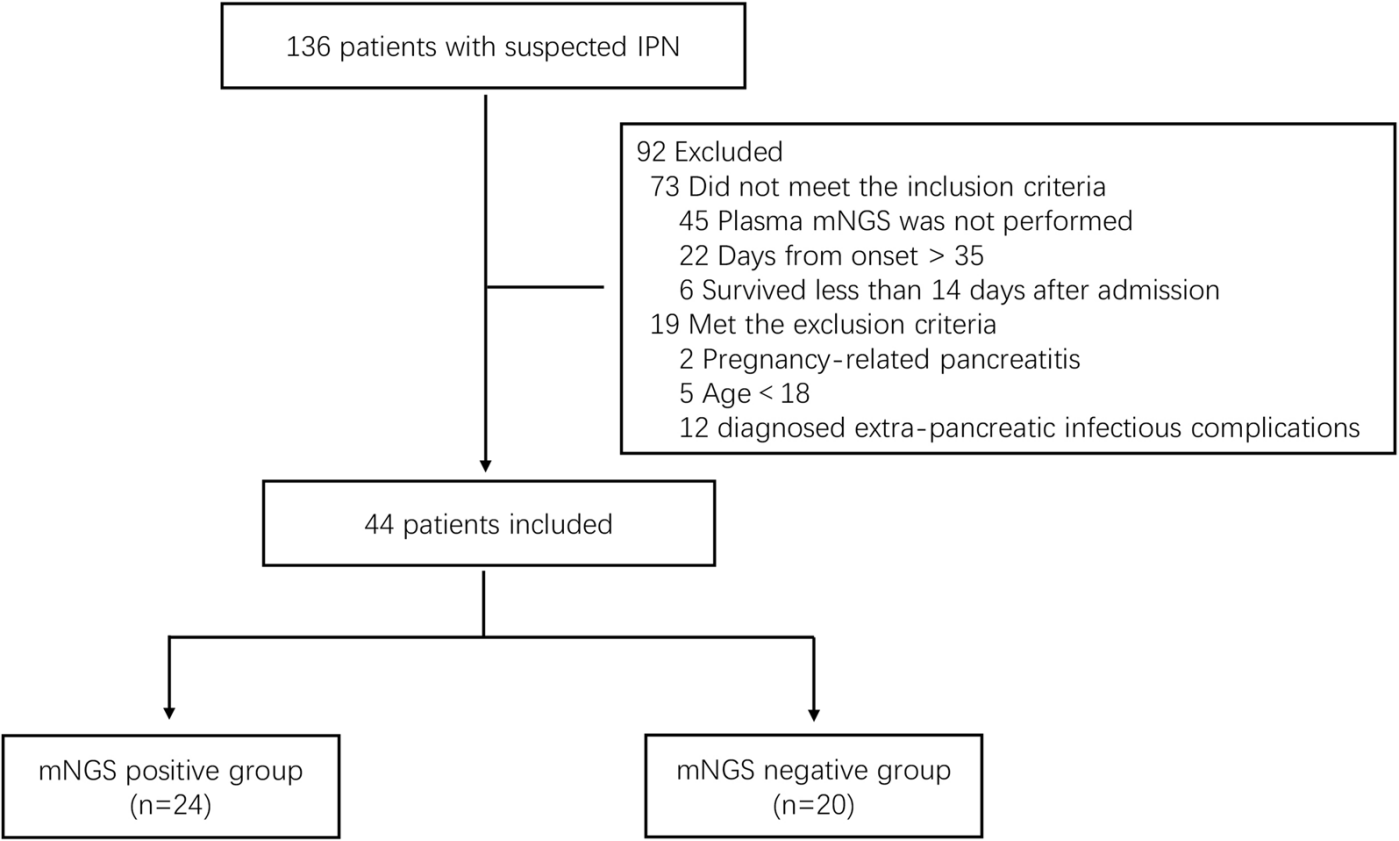
Flow chart of participants included in the trial. *IPN* infected pancreatic necrosis, *mNGS* metagenomic next-generation sequencing

As shown in Fig. 1, forty-four eligible patients were included in this retrospective study and dichotomized into mNGS positive group (n = 24, 54.55%) and mNGS negative group (n = 20, 45.45%). The baseline characteristics were not significantly different between groups in terms of demographics, etiology, and severity of disease (Table 1). The median time from onset to sampling was 22 (16.25–26.75) days, with no difference between the two groups [23.5 (19.25–29.75) vs. 20.0 (14.50–24.50), p = 0.158]. The C-reactive protein levels were significantly different between groups, but no significant difference was observed in procalcitonin and leukocyte count. The use of antibiotics is shown in Additional file 1: Table S1.

**Table 1:** Baseline characteristics

	mNGS positive(n = 24)	mNGS negative(n = 20)	P value
Age (years)	52 (40.25, 62.50)	42.0 (32.0, 58.75)	0.131
Gender (male, %)	18 (75.0%)	13 (65.0%)	0.469
Degree of severity			
Mild	1 (4.2%)	1(5.0%)	0.170
Moderately severe	2 (8.3%)	6 (30.0%)	
Severe	21(87.5%)	13(65.0%)	
Etiology			
Hypertriglyceridemia	9 (37.5%)	11 (55.0%)	0.384
Gallstone	14 (58.3%)	6 (30.0%)	
Other	1 (4.2%)	3 (15.0%)	
APACHE II score	13(10.25, 20.5)	12.50 (7.5, 14)	0.078
SOFA score	4 (2, 7.75)	2 (1.25, 4.75)	0.060
CTSI score	8 (8, 10)	8 (8, 9.5)	0.672
Temperature (°C)	38.8 (38.25, 39.50)	38.45 (38.325, 38.9)	0.143
PCT (μg/L)	1.795 (0.403, 5.155)	0.80 (0.25, 1.67)	0.099
CRP (mg/L)	171.30 (104.38, 240.23)	105.55 (46.70, 153.45)	0.022
Leukocyte (× 10^9^/L)	12.475(7.85, 16.82)	12.07 (7.43, 14.27)	0.396
Neutrophils (× 10^9^/L)	10.75 (9.94, 14.86)	10.26 (6.35, 11.93)	0.525
SIRS	21 (87.5%)	18 (90.0%)	0.589
DM	5 (20.8%)	5 (25.0%)	0.743
Shock	9 (37.5%)	2 (10.0%)	0.036
AKI	8 (33.3%)	3 (15.0%)	0.162
ARDS	13 (54.2%)	7 (35.0%)	0.221
Recurrent AP	3 (12.5%)	1 (5.0%)	0.253
Days from onset (days)	23.5 (19.25, 29.75)	20.0 (14.50, 24.50)	0.158

Data presented as median (interquartile range) or n (%), as appropriate. *mNGS* metagenomic next-generation sequencing, *APACHE II Score* acute physiology and chronic health assessment II score, *SOFA Score* sequential organ failure assessment score, *CTSI Score* computed tomography severity index score, *SIRS* systemic inflammatory response syndrome, *AKI* acute kidney injury, *ARDS* acute respiratory distress syndrome, *PCT* procalcitonin, *CRP* C-reactive protein

### Plasma mNGS results

**Fig. 2:**
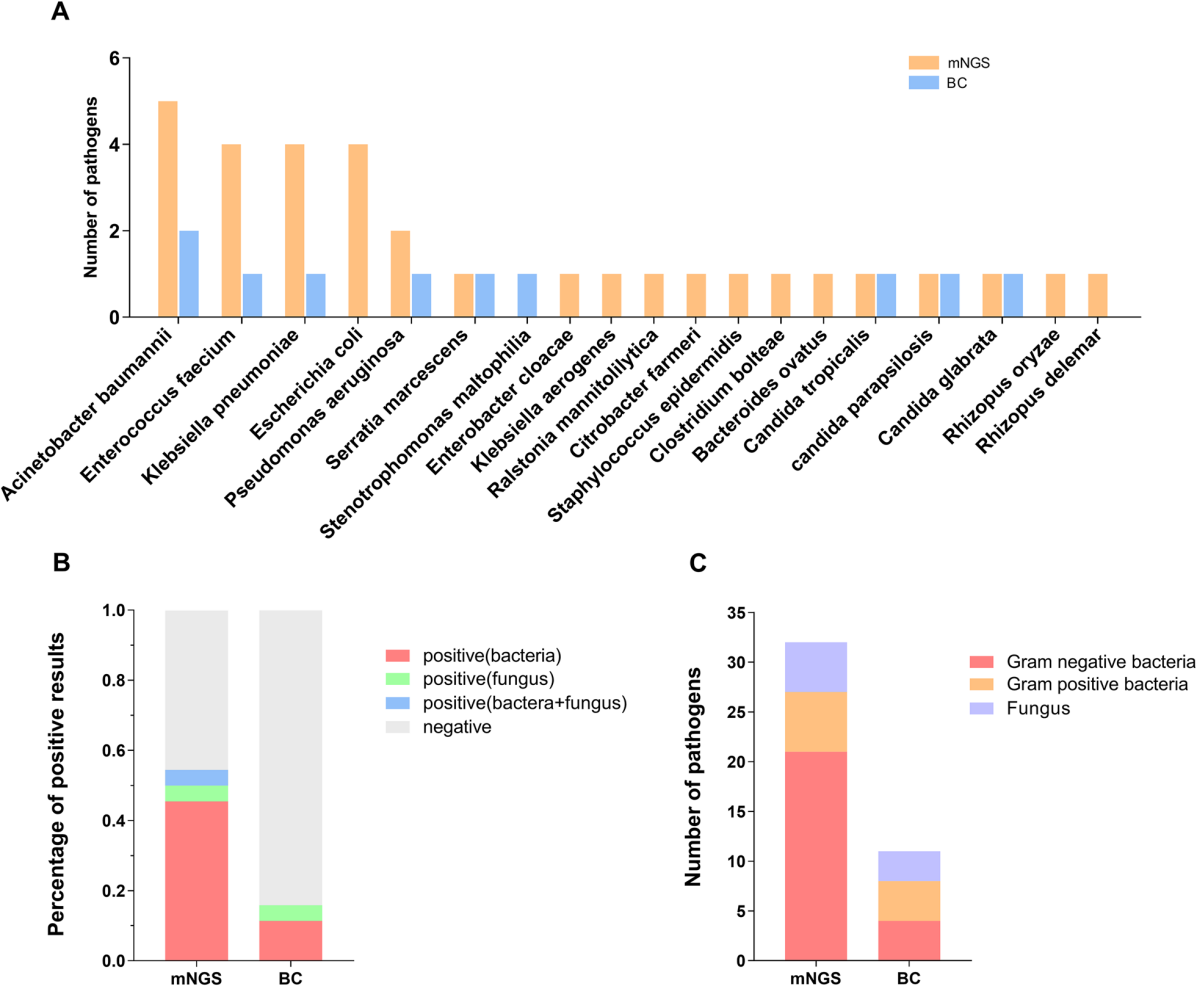
Comparison of plasma mNGS and blood culture for detection of pathogens. **A** Pathogens detected by mNGS and BC; **B** Comparison of positive rates of mNGS and BC. **C** Comparison of the number of pathogens detected of mNGS and BC; *mNGS* metagenomic next-generation sequencing, *BC* blood culture

The results of pathogens detected by plasma mNGS and blood culture are shown (Fig. 2A). Overall, the positive rate of plasma mNGS was much higher than culture [24 (54.55%) vs. 6 (13.64%), p < 0.001] (Fig. 2B). Seven species of bacteria and two species of fungi (9 strains in total) were detected by blood culture. Meanwhile, 13 species of bacteria and 5 species of fungi (32 strains in total) were found by mNGS (Fig. 2C), including rare or difficult-to-culture microbes (such as *Bacteroides ovatus* and *Clostridium bolteae*). In the list of pathogens detected by mNGS, *Acinetobacter baumannii* (n = 5) and was the most common, followed by *Klebsiella pneumoniae* (n = 4), *Enterococcus Faecium* (n = 4) and *Escherichia coli* (n = 4). Six of the mNGS-positive patients were found to have polymicrobial infections. A total of three DNA viral species were identified by plasma mNGS, including human cytomegalovirus (n = 14), herpes simplex virus-1 (n = 2) and Epstein-Barr virus (n = 2). However, we did not conduct traditional virological tests on blood and drainage samples, so we defined samples that detected only viral sequences as mNGS negative. The mNGS data for the pathogens detected are shown in Additional file 1: Table S2, including sequence reads, relative abundance, genome coverage and depth.

In addition, the median time from sampling to report was 73.50 (71–85 h) for the culture approach and 46.50 (43.25–48.00 h) for the mNGS approach, respectively (p < 0.001).

### Tests performance of plasma mNGS in detecting IPN related pathogens

**Fig. 3:**
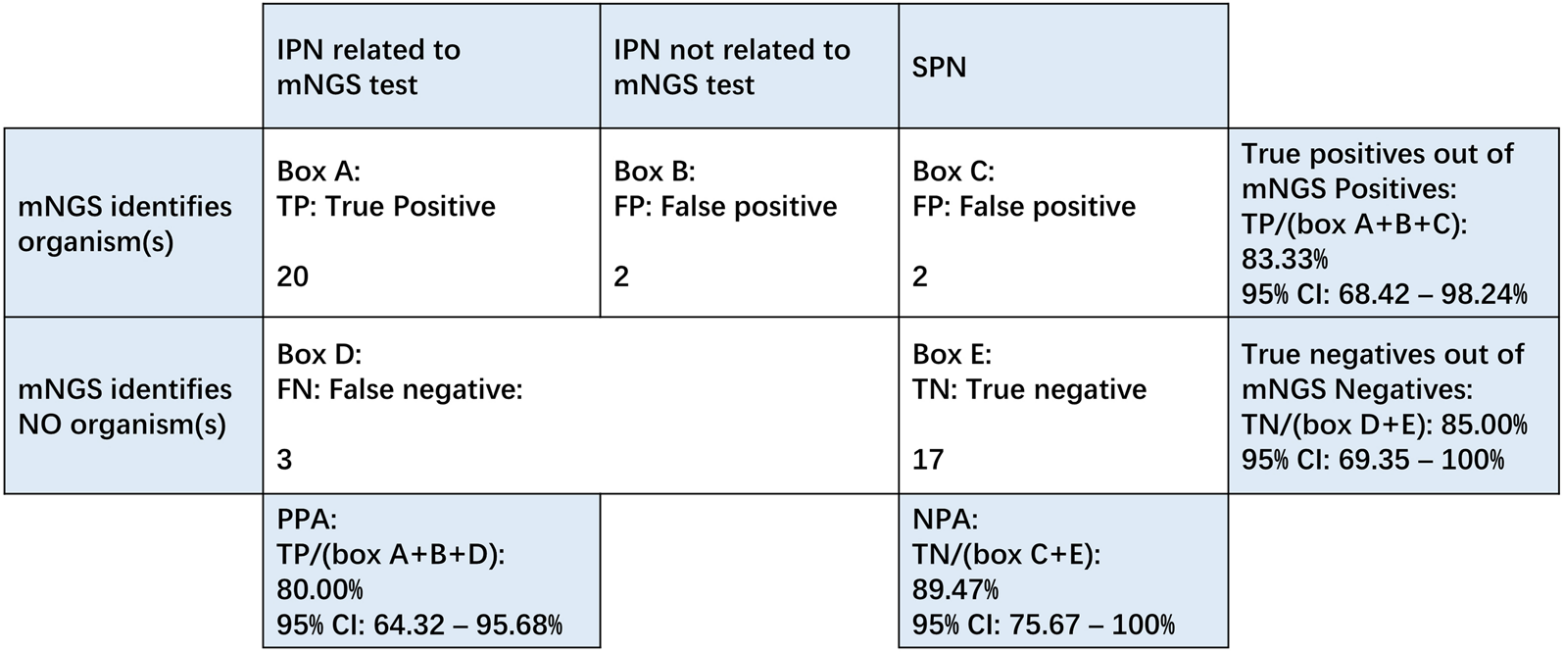
Tests performance of plasma mNGS in detecting IPN related pathogens. *IPN* infected pancreatic necrosis, *SPN* sterile pancreatic necrosis, *PPA* positive percent agreement, *NPA* negative percent agreement

Twenty-nine patients underwent percutaneous catheter drainage (PCD) and microbial culture within the following two weeks after the plasma mNGS. Twenty-five (56.82%) of the study subjects developed microbiologically confirmed IPN within two weeks after sampling, and 22 (91.67%, 22/24) were in the mNGS positive group. Table 2 shows the results of (peri)pancreatic drains culture and plasma mcfDNA in IPN cases. Of the 24 positive mNGS tests, 20 (83.33%, 95% CI 68.42–98.24%) were considered IPN relevant and were considered to be true positive. The PPA and NPA of plasma mNGS are 80.0% (20/25; 95% CI, 64.32–95.68%) and 89.47% (17/19; 95% CI, 75.67–100%), respectively (Fig. 3). Of the four cases of false-positive (Box B and Box C, Fig. 3), circulating mcfDNA were found to be associated with cholecystitis or ventilator-associated pneumonia. In addition, we found three false-negative cases (ID: P1, P46, P76).

**Table 2:** Comparison of Plasma mNGS and (peri)pancreatic drains culture

ID	Plasma mNGS	(Peri)pancreatic drains culture	Interval time (days)	PCD from AP onset (days)
P1	Negative	*A. baumannii*	12	21
P6	*E. faecium*	*E. faecium*	3	33
P7	*K. pneumoniae*	*K. pneumoniae, C. tropicalis*	5	25
P11	*E. coli*	*E. coli*	3	15
P13	*P. aeruginosa, E. coli*	*E. coli*	5	35
P15	*Rhizopus oryzae, Rhizopus delemar*	*P. mirabilis, M. morganii*	10	21
P18	*A. baumannii*	*A. baumannii*	3	23
P21	*E. cloacae, Citrobacter freundii*	*E. cloacae,*	10	32
P22	*Ralstonia mannitolilytica*	*Ralstonia mannitolilytica*	7	29
P29	*E. coli*	*E. coli*	3	27
P32	*E. faecium*	*E. faecium*	2	19
P34	*A. baumannii*	*A. baumannii*	6	21
P39	*K. pneumoniae, C. glabrata*	*C. glabrata*	4	20
P41	*Bacteroides ovatus, Clostridium bolteae*	*E. faecium*	4	22
P46	Negative	*A. baumannii*	9	27
P48	*S. epidermidis*	*S. epidermidis*	4	30
P49	*E. faecium*	*E. faecium*	3	41
P54	*K. pneumoniae*	*K. pneumoniae*	10	24
P61	*K. aerogenes*	*K. aerogenes, E. faecium*	6	28
P63	*E. coli*	*E. coli, K. aerogenes*	7	21
P67	*K. pneumoniae*	*K. pneumoniae*	4	24
P75	*A. baumannii, C. parapsilosis*	*A. baumannii*	14	32
P76	*Negative*	*K. pneumoniae*	4	21
P78	*A. baumannii, P. aeruginosa*	*A. baumannii*	10	21
P83	*C. tropicalis*	*C. tropicalis*	10	23

Interval time: days between sampling and IPN diagnosis. *E. faecium*: *Enterococcus faecium*, *K. pneumoniae*: *Klebsiella pneumoniae*, *C. tropicalis*: *Candida tropicalis*, *E. coli*: *Escherichia coli*, *P. aeruginosa*: *Pseudomonas aeruginosa*, *C. glabrata*: *Candida glabrata*, *C. parapsilosis*: *Candida parapsilosis*, *S. epidermidis*: *Staphylococcus epidermidis*, *K. aerogenes*: *Klebsiella aerogenes*, *P. mirabilis*: *Proteus mirabilis*, *M. morganii*: *Morganella morganii*

### The clinical outcome between mNGS-positive and -negative groups

Table 3 shows the clinical outcomes of the mNGS positive and negative groups. Compared with the negative group, patients in the positive group had more new-onset sepsis shock (12 (50.0%) vs. 4 (20.0%), p = 0.039). Patients in the mNGS positive group needed more PCD and were more likely to receive surgical intervention. Length of hospital stay (LOS), in-hospital mortality, new-onset sepsis, and organ failure did not differ significantly across the two groups.

**Table 3:** Comparison of the clinical outcome of mNGS-positive and -negative group

	mNGS positive (n = 24)	mNGS negative (n = 20)	P value
LOS (days)	39 (20.75, 58.75)	23.0 (21, 45)	0.140
ICU admission	21 (87.5%)	15 (75%)	0.436
In-hospital mortality	7 (29.2%)	4 (20.0%)	0.484
Invasive intervention			
Numbers of PCD	2 (1, 3)	0 (0, 1)	0.004
Requiring of PEN	4 (16.7%)	1 (5.0%)	0.225
Requiring of ON	8 (33.3%)	1 (5.0%)	0.020
Gastrointestinal fistulas	4 (16.7%)	1 (5.0%)	0.225
Abdominal bleeding	6 (25.0%)	3 (15.0%)	0.413
New-onset sepsis	12 (50.0%)	5 (25.0%)	0.090
New-onset septic shock	12 (50.0%)	4 (20.0%)	0.039
New-onset organ failure	11 (45.8%)	4 (20.0%)	0.072

Data presented as median (interquartile range) or n (%), as appropriate. *LOS* length of hospital stay, *PEN* percutaneous endoscopic necrosectomy; *PCD* percutaneous catheter drainage, *ON* open necrosectomy

## Discussion

This study outlines an institutional experience applying plasma mNGS to patients with suspected IPN. We demonstrated that plasma mNGS could accurately identify pathogens in patients with suspected IPN.

Our study led to a conclusion similar to those demonstrated by previous studies that plasma mNGS has a significantly higher sensitivity to detect pathogens compared with blood culture [14, 26]. These findings are consistent with the natural technical advantage of the mNGS approach since it could detect a broader array of potentially infectious agents [27, 28]. Another possible explanation is that mNGS was less affected by antibiotics, as its tested object, mcfDNA, is retained in circulation for longer [29].

The main source of circulating mcfDNA is from microbial cells or their components that enter the bloodstream through the epithelial mucosa of organs [30, 31]. The plasma mNGS approach is used to diagnose potential infections and identify possible pathogens by capturing and identifying these circulating mcfDNA [15]. Hematogenous translocation of pathogens to (peri)pancreatic tissue is one of the mechanisms of IPN [32, 33]. The positive mNGS results we see in our cohort were largely related to IPN. Hence, the plasma mNGS approach could potentially facilitate antibiotics adjustment or necessary invasive interventions.

Recently, plasma mNGS has become a reliable test for predicting clinically-relevant infections. Goggin et al. [34] performed plasma mcfDNA sequencing of blood samples from 47 patients with recurrent or refractory cancer to predict the occurrence of bloodstream infection (BSI). They found that the sensitivity and specificity of the plasma mcfDNA sequencing test in predicting BSI were 75% (95% CI, 51–90%), and 82% (95%CI, 66–91%), respectively. Wilke et al. [23] retrospectively described 110 subjects who underwent plasma mNGS due to clinical symptoms suggestive of infection, focal imaging finding, immunocompromised or other causes, and the results suggested that compared with conventional tests, the mNGS approach have a PPA of 89.6%, but the NPA was only 52%. Taken together, it is clear that the efficacy of plasma mNGS in detecting pathogens varies across different disease populations. The value of plasma mNGS in diagnosing IPN needs to be further assessed.

Plasma mNGS is vulnerable to multiple confounding factors, such as contamination, background microorganisms, and non-pathogenic microbes, potentially resulting in false positives [35]. Moreover, circulating mcfDNA may come from different infection sites, so the interpretation of mNGS positive results should be combined with clinical manifestations, and if necessary, specific site sample testing should be carried out to confirm the results [15]. In our study, the false-positive cases were considered to be related to extra-pancreatic infection, including cholecystitis or ventilator-associated pneumonia. The false-negative cases may be due to the direct transfer of pathogens from the intestine to the (peri)pancreatic tissue without entering the circulation [36, 37], or to pathogen sequence reads that do not meet the threshold for a positive diagnosis. Therefore, the results of plasma mNGS in this specific cohort should be interpreted with caution.

This study had several limitations. First of all, selection bias is inevitable due to its retrospective nature. Secondly, the relatively small sample size may lead to a reporting bias. Third, the value of virus sequences detected by plasma mNGS has not been evaluated. Finally, no orthogonal laboratory testing was conducted to validate the inconsistent results of culture and NGS, which may decrease the credibility of the results.

## Conclusion

In conclusion, plasma mNGS can accurately identify the pathogens of IPN, potentially enabling more timely and appropriate treatment. Further research is necessary to verify its clinical value in infected pancreatic necrosis.

## Supplementary Information

**Additional file 1. Table S1**. The details of antibiotic use.** Table S2**. The sequence data of plasma mNGS.

## Data Availability

The Data of this manuscript are available at http://ngdc.cncb.ac.cn, reference number PRJCA008208. Further inquiries can be directed to the corresponding author.
