# Supplementary material for: Plasma metagenomic next-generation sequencing of microbial cell-free DNA detects pathogens in patients with suspected infected pancreatic necrosis
Source: BMC Infect Dis. 2022 Aug 5;22:675. doi: 10.1186/s12879-022-07662-2 (PMC9356476; doi:10.1186/s12879-022-07662-2)
Supplement: Supplementary file 1 — Additional file 1. Table S1. The details of antibiotic use. Table S2. The sequence data of plasma mNGS. [file 12879_2022_7662_MOESM1_ESM.docx]

Table S1. The details of antibiotic use

| **ID** | **Antibiotics before sampling** | **Antibiotics adjusted after plasma mNGS** | **Antibiotics adjusted after PCD** |
| --- | --- | --- | --- |
| P01 | Cefotaxime | Not adjusted | Cefoperazone Sulbactam |
| P03 | Cefotaxime | Not adjusted | Not applicable |
| P06 | Biapenem | Teicoplanin | Not adjusted |
| P07 | Biapenem | Not adjusted | Biapenem, Caspofungin |
| P10 | Cefotaxime | Not adjusted | Not applicable |
| P11 | Biapenem | Not adjusted | Not adjusted |
| P13 | Cefoperazone Sulbactam | Biapenem | Not adjusted |
| P14 | Cefoperazone Sulbactam | Not adjusted | Not applicable |
| P15 | Cefotaxime | Biapenem, Caspofungin | Not adjusted |
| P17 | Cefotaxime | Not adjusted | Not applicable |
| P18 | Biapenem | Tigecycline | Not adjusted |
| P19 | Not used | Not used | Not used |
| P20 | Cefotaxime | Not adjusted | Not adjusted |
| P21 | Biapenem | Not adjusted | Not adjusted |
| P22 | Biapenem | Not adjusted | Not adjusted |
| P24 | Cefoperazone Sulbactam | Not adjusted | Not applicable |
| P28 | Cefotaxime | Not adjusted | Not applicable |
| P29 | Biapenem | Not adjusted | Not adjusted |
| P32 | Biapenem, Teicoplanin | Not adjusted | Not adjusted |
| P33 | Cefoperazone Sulbactam | Not adjusted | Not applicable |
| P34 | Biapenem | Tigecycline | Not adjusted |
| P38 | Not used | Not used | Not applicable |
| P39 | Biapenem | Tigecycline, Caspofungin | Not adjusted |
| P40 | Not used | Not used | Not applicable |
| P41 | Biapenem | Not adjusted | Not adjusted |
| P42 | Cefoperazone Sulbactam | Not adjusted | Not adjusted |
| P45 | Biapenem | Not adjusted | Not adjusted |
| P46 | Cefotaxime | Not adjusted | Cefoperazone Sulbactam |
| P47 | Cefotaxime | Not adjusted | Not adjusted |
| P48 | Cefotaxime | Teicoplanin | Not adjusted |
| P49 | Cefotaxime | Teicoplanin | Not applicable |
| P50 | Not used | Not used | Not applicable |
| P54 | Cefotaxime | Tigecycline, Biapenem | Not adjusted |
| P56 | Cefotaxime | Not adjusted | Not applicable |
| P59 | Cefotaxime | Not adjusted | Not applicable |
| P61 | Cefotaxime | Biapenem | Teicoplanin, Biapenem |
| P63 | Cefotaxime | Biapenem | Tigecycline |
| P66 | Not used | Not used | Not applicable |
| P67 | Cefoperazone Sulbactam | Tigecycline, Biapenem | Not adjusted |
| P75 | Cefoperazone Sulbactam | Cefoperazone Sulbactam, Caspofungin | Not adjusted |
| P76 | Biapenem | Not adjusted | Tigecycline |
| P78 | Cefoperazone Sulbactam | Not adjusted | Not adjusted |
| P83 | Biapenem | Cefoperazone Sulbactam, Caspofungin | Not adjusted |
| P84 | Not used | Not used | Not applicable |

mNGS: metagenomic next-generation sequencing; PCD: percutaneous catheter drainage; Not applicable: the patient has not received PCD.

Table S2. The sequence data of plasma mNGS

| ID | Raw data size(bp) | human-derived genes(%) | Pathogenic Microorganisms | Sequences reads | Relative abundance | Genome coverage | Depth (X) |
| --- | --- | --- | --- | --- | --- | --- | --- |
| P01 | 6573036 | 99.95 |  |  |  |  |  |
| P03 | 8507697 | 99.86 |  |  |  |  |  |
| P06 | 173981067 | 99.75 | *Enterococcus faecium* | 506 | 0.87% | 1.40% | 1.35 |
| P07 | 3468797 | 99.95 | *Klebsiella pneumoniae* | 126 | 37.99% | 0.17% | 1.05 |
| P10 | 21422402 | 99.9 |  |  |  |  |  |
| P11 | 7172399 | 99.74 | *Escherichia coli* | 2455 | 19.25% | 6.85% | 1.08 |
| P13 | 5184695 | 99.82 | *Pseudomonas aeruginosa* | 20 | 1.82% | 0.02% | 1.02 |
|  |  |  | *Escherichia coli* | 14 | 1.72% | 0.04% | 1.03 |
| P14 | 71253158 | 99.88 | *Enterococcus faecium* | 37 | 0.32% | 0.15% | 1.07 |
| P15 | 36688209 | 99.93 | *Rhizopus oryzae* | 200 | 0.42% | 0.05% | 1.04 |
|  |  |  | *Rhizopus delemar* | 24 | 0.04% | 0.01% | 1.06 |
| P17 | 20983459 | 99.95 |  |  |  |  |  |
| P18 | 16743550 | 99.93 | *Acinetobacter baumannii* | 306 | 16.91% | 0.68% | 1.06 |
| P19 | 29792850 | 99.23 |  |  |  |  |  |
| P20 | 12123201 | 99.96 |  |  |  |  |  |
| P21 | 31361448 | 99.9 | *Citrobacter farmeri* | 31 | 0.26% | 0.10% | 1.09 |
|  |  |  | *Enterobacter cloacae* | 81 | 1.14% | 0.10% | 1.08 |
| P22 | 27601990 | 99.95 | *Ralstonia mannitolilytica* | 416 | 24.68% | 0.68% | 1.05 |
| P24 | 29054165 | 99.82 |  |  |  |  |  |
| P28 | 31144763 | 99.92 | *Serratia marcescens* | 38 | 1.37% | 0.06% | 1.03 |
|  |  |  | *Acinetobacter baumannii* | 82 | 3.53% | 0.18% | 1.04 |
| P29 | 21668314 | 99.95 | *Escherichia coli* | 1011 | 3.01% | 3.13% | 1.05 |
| P32 | 24625651 | 99.52 | *Enterococcus faecium* | 1005 | 22.47% | 3.56% | 1.04 |
| P33 | 24734629 | 99.64 |  |  |  |  |  |
| P34 | 20355375 | 99.92 | *Acinetobacter baumannii* | 682 | 22.53% | 1.43% | 1.07 |
| P38 | 19978524 | 99.82 |  |  |  |  |  |
| P39 | 64678227 | 99.88 | *Klebsiella pneumoniae* | 40 | 0.45% | 0.06% | 1.05 |
|  |  |  | *Candida glabrata* | 7 | 0.04% | 0.00% | 1.17 |
| P40 | 25938428 | 99.85 |  |  |  |  |  |
| P41 | 27081076 | 99.84 | *Bacteroides ovatus* | 1851 | 8.60% | 5.71% | 1.09 |
|  |  |  | *Clostridium bolteae* | 65 | 0.31% | 0.10% | 1.05 |
| P42 | 16056666 | 98.89 |  |  |  |  |  |
| P45 | 15982578 | 99.91 |  |  |  |  |  |
| P46 | 25142959 | 99.75 |  |  |  |  |  |
| P47 | 16807703 | 99.92 |  |  |  |  |  |
| P48 | 22897104 | 99.92 | *Staphylococcus epidermidis* | *368* | 27.99% | 1.08% | 1.04 |
| P49 | 23162488 | 99.81 | *Enterococcus faecium* | 178 | 7.67% | 0.63% | 1.07 |
| P50 | 22636292 | 99.83 |  |  |  |  |  |
| P54 | 19019670 | 99.94 | *Klebsiella pneumoniae* | 22 | 6.94% | 0.03% | 1.01 |
| P56 | 19602074 | 99.34 |  |  |  |  |  |
| P59 | 15132757 | 99.95 |  |  |  |  |  |
| P61 | 16452876 | 99.8 | *Klebsiella aerogenes* | 1445 | 19.41% | 2.10% | 1.07 |
| P63 | 32999320 | 99.91 | *Escherichia coli* | 448 | 11.53% | 1.04% | 1.04 |
| P66 | 5487610 | 99.95 |  |  |  |  |  |
| P67 | 32434846 | 99.98 | *Klebsiella pneumoniae* | 314 | 11.38% | 0.44% | 1.03 |
| P75 | 20040206 | 99.95 | *Candida parapsilosis* | 8 | 0.43% | 0.00% | 1.04 |
|  |  |  | *Acinetobacter baumannii* | 50 | 8.14% | 0.09% | 1.09 |
| P76 | 19429633 | 99.96 |  |  |  |  |  |
| P78 | 18915040 | 99.03 | *Pseudomonas aeruginosa* | 125 | 0.95% | 0.15% | 1.08 |
|  |  |  | *Acinetobacter baumannii* | 153 | 1.68% | 0.33% | 1.09 |
| P83 | 9908529 | 99.97 | *Candida tropicalis* | 22 | 0.59% | 0.01% | 1.11 |
| P84 | 5478937 | 99.94 |  |  |  |  |  |

This table only shows the sequence parameters of pathogens that meet the positive criteria, while data for background microorganisms and viruses can be consulted in the raw data (http://ngdc.cncb.ac.cn).
